# Quick Multi-Class Determination of Residues of Antimicrobial Veterinary Drugs in Animal Muscle by LC-MS/MS

**DOI:** 10.3390/molecules23071736

**Published:** 2018-07-16

**Authors:** Meiyu Zhang, Erfen Li, Yijuan Su, Yingxia Zhang, Jingmeng Xie, Limin He

**Affiliations:** 1 National Reference Laboratory of Veterinary Drug Residues (SCAU), College of Veterinary Medicine, South China Agricultural University, Guangzhou 510642, China; zmy19900210@163.com (M.Z.); syj@scau.edu.cn (Y.S.); 2 Guangdong Provincial Key Laboratory of Veterinary Pharmaceutics, Development and Safety Evaluation, South China Agricultural University, Guangzhou 510642, China; 13424456269@163.com (E.L.); zhangyx215303@163.com (Y.Z.); xjm030527@163.com (J.X.)

**Keywords:** veterinary drugs, generic extraction solvent, matrix effects, liquid chromatography-tandem mass spectrometry, dilution strategy, animal muscle

## Abstract

On the basis of the highly sensitive and selective liquid chromatography-tandem mass spectrometry technique, a generic extraction solvent and a sample dilution method was developed for the residue analysis of different polar veterinary drugs known as fluoroquinolones, sulfonamides, macrolides, and tiamulin in chicken muscle. The results showed that the matrix-matched calibration curves of all 10 compounds were in an effective linear relationship (*r*^2^ ≥ 0.997) in the range of 0.2–100 μg L^−1^. At three spiking levels of 2 (5), 50, and 100 μg kg^−1^, average recoveries of analytes were between 67.1% and 96.6% with relative standard deviations of intra-day and inter-day below 20%. The limits of detection and limits of quantification of the method were in the range of 0.3–2.0 μg kg^−1^ and 2.0–5.0 μg kg^−1^, respectively, which were significantly lower than their maximum residue limits. In addition, the intensity of the target analytes and its corresponding matrix effects were obviously related to the sample dilution times (matrix concentration). There were no significant differences (*p* > 0.05) in the average content of almost any of the analytes in medicated chickens between this method and the method in the literature for determining analytes. Lastly, the proposed method was successfully applied for the simultaneous analysis of 10 common veterinary drugs in food animal muscle tissues.

## 1. Introduction

Veterinary drugs such as fluoroquinolones, sulfonamides, macrolides, pleuromutilins, β-lactams, aminoglycosides, and tetracyclines are widely used for preventing and treating diseases in animal husbandry [1]. Additionally, some of the drugs such as macrolides, polypeptides, tetracyclines, etc. are licensed for use as feed additives to increase the conversion rate of feed and promote animal growth in China, the United States, and some other countries and regions [2,3]. Although veterinary drugs play an important role in the production of livestock and poultry, their residues in meat products, which are caused by irrational use or over-use, can cause allergic reactions in hypersensitive individuals and destruct the dynamic balance of gastrointestinal flora and increase the microbial antibiotic resistance and the ecological toxicity [4,5,6]. Accordingly, maximum residue limits (MRLs) of many veterinary drugs have been regulated for ensuring animal-derived food safety in Bulletin 235 of the Chinese Ministry of Agriculture (MOA) [7] and the Commission Regulation No. 37/2010 of the European Union (EU) [8]. Therefore, it is of great significance to develop simple, quick, and reasonable methods for the simultaneous determination of residues of multi-class veterinary drugs with set MRLs in animal-derived food.

As we all know, due to the inherent limit of sensitivity of the high-performance liquid chromatography (HPLC) technique, the conventional sample preparation methods usually have to adopt a concentration/enrichment strategy for satisfying the requirements of analysis of veterinary drug residues [9,10,11,12]. Therefore, increasing sample weight and/or reducing the volume of the final sample solutions are usually incorporated. In practice, when the analytes are further enriched, a large number of endogenous impurities are also simultaneously concentrated, which usually leads to poor reproducibility, shortens the lifespan of the chromatographic column, and increases the frequency of the HPLC system maintenance. Certainly, various clean-up techniques such as liquid-liquid extraction [13], solid-phase extraction (SPE) [14,15,16], dispersive solid-phase extraction (DSPE) [17,18], and more are also used. However, any clean-up procedure is a double-edged sword. While it removes a great deal of matrix impurities, the targets are also somewhat lost. Moreover, the clean-up approaches (e.g., SPE) not only increase pre-treatment time and analytical cost, but also hardly get a proper SPE sorbent material for purifying multi-class veterinary drugs with different physical and chemical properties [19]. Unfortunately, with the emergence of a variety of sensitive advanced instruments such as gas chromatography-tandem mass spectrometry (GC-MS/MS) and liquid chromatography-tandem mass spectrometry (LC-MS/MS), the conventional idea behind the concentration of samples is still widely adopted in the residue analyses [20,21,22,23,24]. It is well known that the concentration and enrichment of samples greatly increase matrix effects (MEs) when using tandem mass spectrometry analysis. Matrix effects seriously influence the target’s ionization in LC-MS/MS analysis, which leads to an inadequate accuracy and large deviations.

Based on the description above, we propose a new sample preparation strategy known as the generic extraction solvent plus sample dilution method for the multi-residue analysis of veterinary drugs with MRLs by using LC-MS/MS in a selected reaction monitoring (SRM) mode. Due to LC-MS/MS techniques with high sensitivity and selectivity, as long as veterinary drugs with set high MRL values are concerned, the conventional concentration and enrichment of samples are unnecessary. Conversely, the most reasonable approach is to use the dilution strategy of samples, which can meet the requirements of residue analyses and decrease matrix effects. As a result, the accuracy and precision of the method are enhanced and the frequency of instrument maintenance reduces. Granelli et al. developed a new approach for simple and rapid multi-residue screening of antibiotics in muscle and kidney tissues by LC-MS/MS [25]. Although the dilution strategy of samples was adopted, the extraction solvent of 70% methanol (MeOH) was not an ideal generic extraction solution [21]. There are many co-extract interferences from complex biological matrices when the polar solvent of MeOH is used.

In this paper, we first took 10 common veterinary drugs with set MRLs (enrofloxacin (ENR), ciprofloxacin (CIP), sarafloxacin (SAR), sulfamonomethoxin (SMM), sulfadimidine (SM2), sulfaquinoxaline (SQ), tilmicosin (TIM), tylosin (TYL), kitasamycin (KIT), and tiamulin (TAM)). A generic extraction solvent was used to extract these analytes from chicken muscle samples and the extracting solutions were diluted prior to LC-MS/MS analysis. Therefore, a simple, rapid, and reliable multi-residue analytical method based on LC-MS/MS was established for the simultaneous determination of 10 veterinary drug residues in chicken muscle samples. In the meantime, matrix effects were systematically evaluated. Lastly, the residual amounts of the antimicrobials in two medicated chickens were determined by using the proposed method and the method reported in the literature. The proposed method might be used for the monitoring of common veterinary drug residues in food animal muscle tissues.

## 2. Results and Discussion

### 2.1. Optimization of LC-MS/MS Conditions

LC-MS/MS is the most commonly used technique for the simultaneous determination of multi-class veterinary drug residues because of its high sensitivity, selectivity, and reliable analysis of substances at trace and ultra-trace levels in complex matrices. In this study, the LC-MS/MS instrumental conditions were optimized for obtaining the maximum response of analytes.

#### 2.1.1. Optimization of MS/MS Parameters

The selection of the precursor and product ions is a crucial first step for developing an LC-MS/MS method. An individual standard solution (ca 1 μg mL^−1^) diluted with HPLC-grade methanol was directly infused into the mass spectrometer with a syringe pump at 10 μL min^−1^. In the positive mode, the experimental results showed that each compound could be efficiently ionized and had higher sensitivity. In compliance to the 2002/657/EC requirements (IPs ≥ 4), the optimum parameters for analytes were selected to obtain two selected reaction monitoring (SRM) transitions. The high intensity transition used the quantitative ion and the identification ion. The optimized mass spectrum parameters of all target analytes were shown in Table 1.

#### 2.1.2. Optimization of Chromatographic Conditions

Several different brands of C_18_ reversed-phase columns were used to separate the 10 antibiotics. Superior chromatographic behavior target analytes when using the Phenomenex Luna C_18_ column instead of other reversed-phase columns. In this study, 0.05% formic acid in acetonitrile (ACN)-0.05% formic acid in water and 0.05% formic acid in MeOH-0.05% formic acid in water were tested during the mobile phase system. The results showed that using 0.05% formic acid in ACN-0.05% formic acid in water had a better mobile phase, response, peak shape, and separation for target analytes than the mobile phase of 0.05% formic acid in MeOH-0.05% formic acid in water. Consequently, the system of 0.05% formic acid in ACN-0.05% formic acid aqueous solution was selected as the mobile phase.

### 2.2. Optimization of Sample Preparation

#### 2.2.1. Screening of the Generic Extraction Solvent

**Figure 1:**
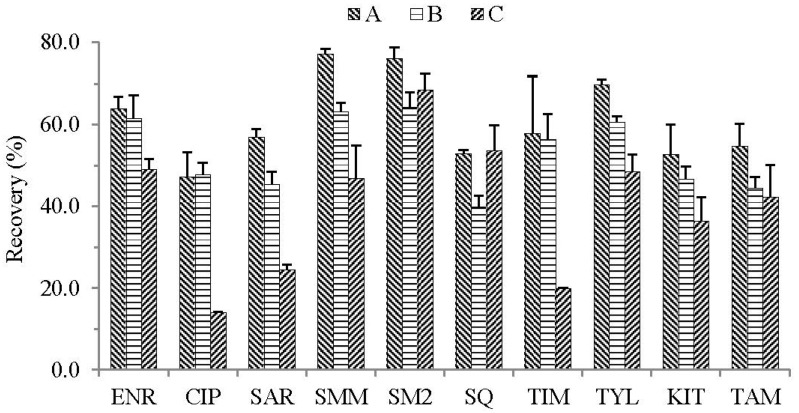
Influence of different extraction solvents on the recoveries of compounds in the chicken muscle sample at the spiked 50 µg kg^−1^ (*n* = 5), (A) Acetonitrile, (B) Methanol, (C) Ethyl acetate.

In the present study, we put hypothesized that, in order to find a generic extraction solvent that could effectively extract 10 common veterinary drugs from food animal muscle matrixes and minimize matrix co-extract impurities, the extracts need to be subjected to proper dilution for LC-MS/MS analysis. Therefore, 10 commonly used and different polar veterinary drugs including ENR, CIP, SAR, SMM, SM2, SQ, TIM, TYL, KIT, and TAM were used. On the basis of the literature [26,27], several common extraction solvents such as ACN, MeOH, and ethyl acetate were tested to extract the target analytes in chicken muscle samples. As shown in Figure 1, when ethyl acetate was used as the extraction solvent, even though the co-extract impurities were less, the average recoveries of the target compounds were less than 55% except for SM2 (68.4%). When using MeOH as the extraction solvent, the average recoveries of all drugs were lower than 65%. Furthermore, more impurities were co-extracted by MeOH [27], which is adverse to LC-MS/MS analysis. When ACN was used as the extraction solvent, the extracting solutions were relatively cleaner and the average recoveries of compounds were slightly higher than MeOH. Nevertheless, the average recoveries of compounds were still less than 70% except SMM (77.1%) and SM2 (75.9%).

**Figure 2:**
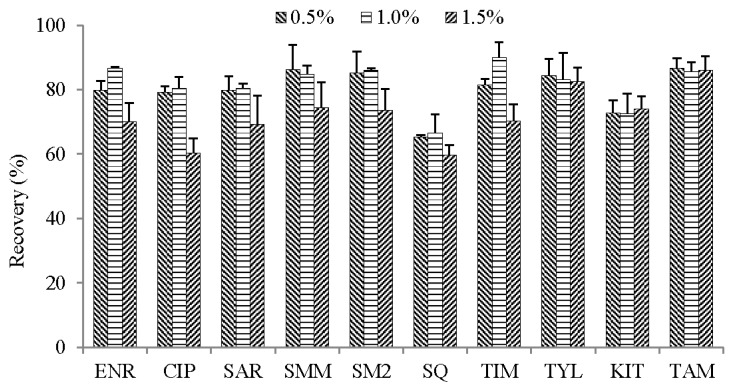
Influence of different percentages of acetic acid in acetonitrile on the recoveries of compounds in the chicken muscle sample at the spiked 50 µg kg^−1^ (*n* = 5).

The addition of acetic acid in the extraction solvent can effectively improve the extraction efficiencies due to amphoteric drugs holding satisfactory solubility in weak acids [23]. Therefore, different concentrations of acetic acid (0.5%, 1% and 1.5%) in ACN were performed to improve the extraction efficiencies of compounds in chicken muscle samples. The results were shown in Figure 2, using 0.5% acetic acid in ACN as the extraction solvent, the extraction efficiencies of analytes ranged from 65.3% to 86.6%. When 1% acetic acid in ACN was used as the extraction solvent, the extraction recoveries of most analytes (66.5–86.6%) were slightly higher than those of 0.5% acetic acid in ACN. The extraction recoveries of fluoroquinolones (ENR, CIP, and SAR) and TIM significantly decreased when using 1.5% acetic acid in can while the remaining analytes showed no clear differences. Therefore, 1% acetic acid in ACN was selected as the extraction solvent.

Afterward, different volumes (5 mL, 10 mL, 15 mL, and 20 mL) of 1% acetic acid in ACN were further optimized. The extraction efficiencies of analytes ranged from 53.6% to 76.2% when using 5 mL of 1% acetic acid in ACN. The extraction efficiencies of the compounds significantly increased (66.4% to 91.8%) when using 10 mL of 1% acetic acid in ACN. In 10 milliliters or more 1% acetic acid in ACN, the extraction recoveries of analytes showed no clear differences. Accordingly, to reduce the use of the organic reagent, 10 mL 1% acetic acid in ACN was chosen as the extraction solvent to conduct the extraction step.

#### 2.2.2. Sample Dilution Strategy

The purpose of this study was to propose a sample pre-treatment strategy for the analysis of residues of the common veterinary drugs with set MRLs. Therefore, after a generic extraction solvent was optimized, different times (0.25, 0.5, 1 and 5, 10 times) of sample dilution were evaluated. The results demonstrated that 5 or 10 times the dilution of the sample could meet the requirements of residue analyses of 10 typical antibiotics. In this study, 10 times the dilution of the sample (1:10 of sample weight to extraction volume, g:mL) was adopted to obtain a more reproducible method performance. The conventional concentration and enrichment of the sample were unnecessary and undesirable. Moreover, the sample dilution strategy also decreased matrix effects and the frequencies of instrumental maintenance.

### 2.3. Method Validation

In this study, one precursor and two product ions of each analyte were monitored for conforming to the 2002/657/EC requirements (IPs ≥ 4). The identification was determined by comparing the ratio of the relative intensity of confirmation and quantification transitions of each analyte in pure solvent and the matrix extraction solution at 10 μg L^−1^. The mean ion ratios for 10 compounds were summarized in Table 1. The results showed that the relative deviation of SAR was 21.3% (relative ion abundance ratio (RIAR) above 20%), the relative deviation of TAM was 16.5% (RIAR above 50%), and, no matter how many RIAR, the relative deviations of other analytes were less than 10%. Therefore, the proposed LC-MS/MS method fulfills the specific requirements of permitted relative intensities of SRM transitions in EC validation criteria [28].

**Figure 3:**
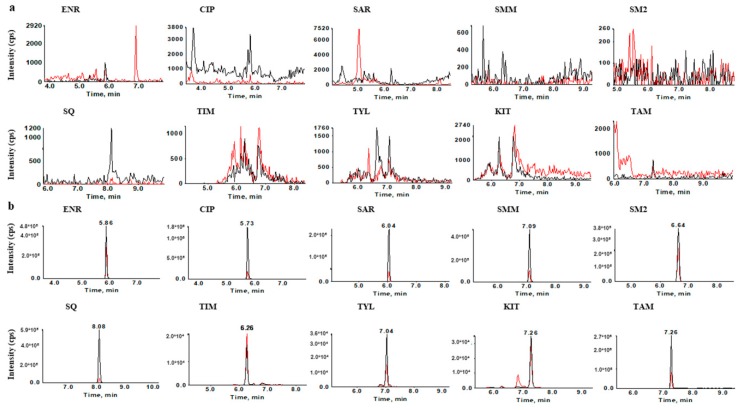
Typical SRM chromatograms of blank chicken muscle extracts (**a**) and its matrix-matched standard (**b**) (10 µg L^−1^). Blank line, quantification ion, red line, confirmation ion.

The selectivity of the method was confirmed by analyzing 20 blank chicken muscle samples and no interfering peaks existed within a 2.5% margin of the retention time of the target compounds. The typical SRM chromatograms of blank chicken muscle extracts and the corresponding matrix-matched standard solution were shown in Figure 3.

The linearity of the method was evaluated with a matrix-matched calibration curve at eight concentration levels in chicken muscle matrixes. In this study, the matrix-matched calibration curves presented good linearity in the range of 0.2 μg L^−1^ to 100 μg L^−1^ for the analytes and correlation coefficients (*r*^2^) values were greater than 0.997.

The accuracy (recovery) and precision of each compound were determined by spiking blank chicken muscle samples at three concentration levels of 2 (5), 50 and 100 μg kg^−1^ (SAR, 2, 10 and 20 μg kg^−1^) with six replicates at each level, respectively. As shown in Table 2, the average recoveries of analytes spiked at the three concentration levels ranged from 67.1% (KIT at 50 μg kg^−1^) to 96.6% (ENR at 50 μg kg^−1^). The intra-day and inter-day relative standard deviations (RSDs) were less than 16% (TAM at 2 μg kg^−1^) and 20% (SAR at 2 μg kg^−1^), respectively, which indicates acceptable precision of the method.

Based on the method described above, the limit of detection (LOD) and the limit of quantification (LOQ) were calculated. The results were summarized in Table 3. The LODs and LOQs ranged from 0.3 μg kg^−1^ to 2.0 μg kg^−1^ and 2.0 μg kg^−1^ to 5.0 μg kg^−1^, respectively. In this developed method, the LOQ values of all compounds in chicken muscle samples were below the requirement values of the Bulletin 235 [7] and the commission regulation [8]. Therefore, the developed method meets the legal requirements of the analysis of veterinary drug residues in chicken muscle.

### 2.4. Matrix Effects

It is well known that there is a severe matrix effect (suppression/enhancement) during LC-MS/MS analysis especially in the ESI mode. The generation of ME is the competition of ionization between the co-eluting interferences and the analytes [29,30]. The ME has a direct effect on the response signal of the analytes resulting in a large deviation in the detection results, which affects the accuracy and precision of the detection method [30,31]. Therefore, elimination or compensation of ME is particularly critical on the establishment of accurate and reliable detection methods.

**Figure 4:**
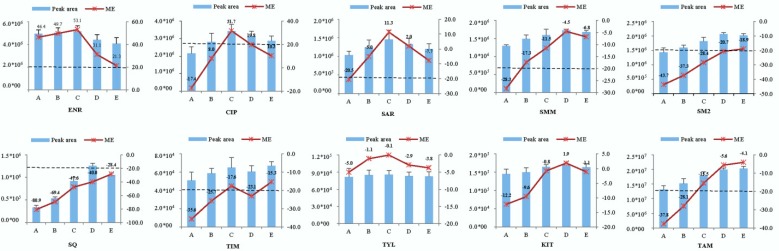
Influence of different sample dilution times on matrix effects of 10 veterinary drugs in chicken muscle matrices at 10 µg L^−1^ (*n* = 20). (A) 0.25 multiple, (B) 0.5 multiple, (C) 1 multiple, (D) 5 multiple, (E) 10 multiple.

In this study, in order to evaluate the feasibility of the proposed method based on the generic extraction solvent and the sample dilution strategy combined with the LC-MS/MS technique, the MEs of analytes were further analyzed in chicken muscle matrices collected from four different sources. The pure solvent (20% MeOH in 0.1% formic acid aqueous solution) and 0.25, 0.5, 1, 5, and 10 times (corresponding to 4:1, 2:1, 1:1, 1:5, and 1:10 of the ratios of sample weight to extraction volume, g:mL) times the dilution of samples were determined. The results were shown in Figure 4. Three sulfonamides (SMM, SM2, and SQ), TIM, and TAM indicated a severe ion suppression phenomenon. For example, the ME of SQ was more than 80% (suppression) when the sample was concentrated four times (0.25 times dilution). There is a significant relationship between the MEs of the five compounds and its corresponding dilution times. With the increase of the sample dilution multiples, the ion suppression effects decreased. However, as for three fluoroquinolones drugs, there were odd MEs phenomena. It caused a high ion signal enhancement effect for ENR and the ME also decreased with the increase of the sample dilution multiples (the ME was about 20% when the dilution multiple was 10). Although there were no severe MEs for CIP and SAR, their MEs showed the first signal suppression and then indicated signal enhancement. The MEs (suppression and enhancement) decreased with an increase of the dilution multiples. Matrix effects are related to various factors such as the concentrations of analytes, matrix components, injection volumes, sample pre-treatment methods, and different ESI structures of LC-MS/MS instruments. Therefore, the unique ME phenomena for CIP and SAR warrant further study. By the way, as for ME of TYL, it was not related to dilution times, but very weak matrix effects (from −5% to −0.1%) were observed within five dilution points.

Strong matrix effects of ion signal suppression or enhancement were observed for most analytes (SMM, SM2, SQ, TIM, TAM, SAR, CIP, and ENR) in the chicken muscle matrix when the sample concentration/enrichment strategy was adopted. Nevertheless, the MEs of all analytes in the chicken muscle matrix reached acceptable range except SQ (−28.4%) and ENR (21.3%) when the sample was diluted to 10 multiples.

### 2.5. Method Application

To verify the proposed method, the residual amounts of 10 antimicrobial drugs in two medicated chickens were determined by the proposed method and the method reported in the literature [32]. All data analyses were performed using SPSS software and the results of analytes determined with the two methods were compared using the independent sample *t*-test at a 95% confidence level (α = 0.05).

**Figure 5:**
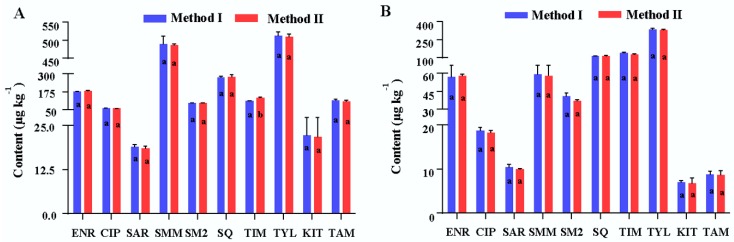
Comparison of the amount of 10 analytes in medicated chickens (**A**) No. 2, (**B**) No. 3 obtained by using the proposed method and the method in literature (*n* = 6). Method I, the method reported in the literature. Method II, the proposed method.

It was shown that the RSDs of 10 analyte amounts in the two medicated chicken samples were less than 15% (six replicates for each chicken) except for KIT in the No. 2 chicken (17.4%). The precision of the proposed method for 10 analytes were acceptable for analysis of veterinary drug residues (all below 20%). The results of an independent sample *t*-test showed that there were no significant differences (*p* > 0.05) between the average values of nine antimicrobials obtained with two methods (See Figure 5). The *p* value of TIM in the No. 2 chicken was 0.000, which indicates that the mean value of TIM obtained with the developed method was significantly greater than that obtained with the method in the literature [32]. At the same time, the analytical methods for single class drug residues in the literature were also selected for determining the analytes in this study. There were also no significant differences (*p* > 0.05) in the average contents of the analytes in medicated chickens between the proposed method and the specific methods [33].

### 2.6. Comparison of the Proposed Method and Method from the Literature

The primary purpose of this study was to find a generic extraction solvent for effectively extracting different polar veterinary drug residues in edible muscle tissues. Therefore, a corresponding method involved in a simple pretreatment approach and 24 important veterinary drugs [32] were selected as controls for determining 10 target analyte residues in the incurred chicken muscle samples after the proposed method was developed. The statistical comparison was evaluated in Section 2.5. In order to further explain the differences of two analytical methods, a detailed comparison was shown in Table 4. Compared with the literature method, acetic acid was replaced of trichloroacetic acid (TCA) in the extraction step, which can avoid the effect of TCA on the ionization efficiency of analytes and reduce routine maintenance of the instrument. Very high speed and a low temperature in the centrifuge step is beneficial for the purification of the sample in the developed method. As mentioned in Section 2.4, the influences of different sample dilution times on MEs were evaluated in this study. The MEs of all analytes in the chicken muscle matrix reached an acceptable range except SQ and ENR. However, strong signal enhancement (higher than 20%) or strong signal suppression (less than −20%) except for streptomycin (4%) were observed in the method reported in the literature [32]. Strong MEs indicated that more impurities were extracted from chicken muscle, which affected the accuracy and precision of the detection method [30].

## 3. Materials and Methods

### 3.1 Reagents and Materials

The standards of ENR, CIP, SAR, SMM, SM2, SQ, TIM, TYL, KIT, and TAM were supplied by the China Institute of Veterinary Drugs Control (Beijing, China). HPLC-grade ACN, MeOH, and formic acid were provided by Fisher Scientific (Fair Lawn, NJ, USA). Analytically pure ACN, MeOH, ethyl acetate, acetic acid, hexane, and TCA were obtained from the Guangzhou Chemical Reagent Company (Guangzhou, China). Ultrapure water was obtained by a Millipore system Milli Q (Molsheim, France).

Stock standard solutions (1 mg mL^−1^) were prepared by dissolving each compound in HPLC-grade methanol or 2% ammonia in methanol (*v/v*) and were then stored at −20 °C in the dark until use. The working standard solutions of compounds at appropriate concentrations were prepared by diluting the individual stock standard solutions in HPLC-grade methanol on the day of use.

### 3.2. Sample Preparation

#### 3.2.1. The Proposed Method

Two grams of homogenized chicken muscle samples were weighed in a 50 mL polypropylene centrifuge tube. Extraction was performed with 10 mL of 1% acetic acid in ACN, which was followed by vortexing for 30 s, an ultrasonic bath for 10 min, and centrifugation at 9000 rpm for 10 min at 4 °C. Afterward, 1 mL of the sample extract solution was evaporated under a gentle stream of nitrogen at 40 °C.

The residues were reconstituted in 2 mL of 20% methanol in water containing 0.1% formic acid. The re-dissolved solution was defatted with 2 mL n-hexane and then centrifuged at 15,000 rpm for 10 min at 4 °C. Lastly, the solutions were filtered through a 0.22 µm filter and analyzed by LC-MS/MS.

#### 3.2.2. The Method Reported in Literature [32]

Two grams of chicken muscle samples were extracted with 10 mL of a solution of 2% TCA aqueous solution: ACN (1:1, *v/v*). The tube was shaken vigorously for 10 min and centrifuged at 8000 rpm for 5 min. One milliliter of supernatant was decanted into a polypropylene centrifuge tube and then 5 mL of hexane was added to remove fat. The mixture was vortexed for 1 min and centrifuged again at 3400 rpm for 5 min. The hexane layer was discarded. A 250 μL of sample extract was diluted with 750 μL of 10% formic acid in water: ACN (1:9, *v/v*). Lastly, the supernatant was filtered through a 0.22 µm syringe filter and analyzed by LC-MS/MS. 

### 3.3. LC-MS/MS Analysis

Chromatographic separation was performed using a Shimadzu liquid chromatography (LC) system consisting of a DGU-20 A 5 R mobile phase degasser unit, two LC-30 AD solvent delivery pumps, SIL-30 AC cooling autosampler, and a CTO-20 A prominence column oven (Shimadzu, Kyoto, Japan). The analytes were separated on a Phenomenex Luna C_18_ reversed-phase column (150 mm × 2.1 mm i.d., 5 μm, Torrance, CA, USA) at 30 °C. The mobile phase consisted of 0.05% formic acid in ACN (A) and 0.05% formic acid in water (B). The flow rate was 0.25 mL min^−1^ and the injection volume was 5 μL. The gradient elution program was as follows: 0–2 min, 10 % A, 2–10 min, 10–90% A, 10–10.5 min, 90–10% A, 10.5–12 min, 10% A. The total run time was 12 min.

Mass spectrometry analyses were carried out by an Applied Biosystems Sciex TRIPLE QUAD 5500 triple-quadrupole mass spectrometer with a syringe pump. The data acquisition was performed by using analyst 1.6.3 software (Foster City, CA, USA). The instrument was operated on using electrospray ionization in a positive ion mode (ESI^+^) for all the analytes. The operation conditions include: ion spray voltage (IS), 5500 V; dwell time, 50 ms; ion source temperature, 600 °C; curtain gas (CUR), 40 psi; auxiliary gas GS1, 55 psi, and auxiliary gas GS2, 55 psi. The optimization of the main mass spectrometric parameters and selected reaction monitoring transitions for each compound were shown in Table 1.

### 3.4. Method Validation

In this work, the developed method for determining veterinary drug residues in chicken muscle was validated through the assessment parameters that meet regulations defined by the European Commission Decision 2002/657/EC [28].

Identification of a target analyte must meet the tolerance for the retention time, identification points (IPs) of the analyte, and the limit of the relative abundance of selected SRM transitions. Identification was determined by the comparison of a relative ion abundance ratio of the confirmation transition and quantification transition of the target analyte in pure solvent (20% methanol in 0.1% formic acid aqueous solution) and blank matrix extract solution at 10 μg L^−1^. The acceptance criterion stipulates that, if RIAR is more than 50%, the maximum permitted tolerance for relative ion intensity (MPTRI) is ±20%. If RIAR ranges from 20% to 50%, MPTRI is ±25%. If RIAR ranges from 10% to 20%, MPTRI is ±30%. If RIAR is less than 10%, MPTRI is ±50%.

Selectivity of the method was validated by analyzing 20 blank chicken muscle samples from different origins in order to verify the existence of the endogenous interferences of each target analyte. The acceptance criterion stipulates that no interfering peaks exist within the 2.5% margin of relative retention time of the target analytes.

The linearity of the developed analytical method was investigated by analyzing the matrix-matched calibration curve constructed by an external standard approach using spiking blank extracts at eight concentrations of 0.2 μg L^−1^, 0.5 μg L^−1^, 1 μg L^−1^, 5 μg L^−1^, 10 μg L^−1^, 20 μg L^−1^, 50 μg L^−1^, and 100 μg L^−1^ for all compounds. External standard matrix-matched calibration curves originated from using the peak area of each analyte versus its corresponding concentration.

Accuracy (recovery) and precision were assessed using chicken muscle samples spiked at three concentration levels of 5 μg kg^−1^, 50 μg kg^−1^, and 100 μg kg^−1^ (SAR, 2, 10 and 20 μg kg^−1^) with six replicates of each concentration on three different days. The recovery was determined by comparing the concentration value measured from the matrix-matched calibration curve and the concentration value spiked in blank chicken samples. The intra-day precision was carried out within a day while the inter-day precision was evaluated on three different days. The intra-day and inter-day precisions were expressed as the RSD.

LOD and LOQ of the method were defined as the lowest concentration of the analytes that can be recognized/quantified by the detector with a signal-to-noise ratio higher than 3 and 10 times in the matrix, respectively.

### 3.5. Matrix Effects

The matrix effect was evaluated by comparing the average peak area of an analyte in four different chickens with five replicates of each chicken in the matrix-matched standard (A) and the pure solution (B). The equation of ME includes the following: ME (%) = (A/B−1) × 100 [34]. The ME ranging from −20% to 20% indicates that the matrix effect can be accepted while less than −20% indicates a strong ion suppression effect and more than 20% indicates a strong signal enhancement effect [35].

### 3.6. Method Application

The study used 3 healthy chickens with an average weight of 1.0 ± 0.1 kg. They were fed without any antimicrobial additives for a week. One chicken was randomly selected as the blank control group and the other two chickens received nine antimicrobials. Dosage, time of administration, and time point of slaughter [36,37,38,39] are shown in Table 5.

## 4. Conclusions

A new generic solvent and sample dilution strategy was created to extract the residues of 10 veterinary drugs with maximum residue limits in chicken muscle samples. Based on this, a quick, practical, and reliable LC-MS/MS method was developed and validated for the simultaneous determination of the multi-class veterinary drug residues. The proposed method could completely satisfy the routine monitoring of the residues of 10 different polar veterinary drugs in animal muscle tissues and reduce the frequency of LC-MS/MS maintenance.

## Figures and Tables

**Table 1:** LC-MS/MS conditions for 10 analytes in the SRM positive ion mode.

Analyte	Precursor	Product	DP ^b^ (V)	CE ^c^ (eV)	Ion Ratio ^d^ (RSD ^e^, %) (*n* = 10)		Relative
	Ion	Ion			Pure Solvent	Muscle Matrix	Deviation (%)
ENR	360.6	316.4 ^a^	60	30	52.6 (0.8)	55.7 (1.2)	5.9
		245.1	60	37			
CIP	332.4	314.2 ^a^	60	25	25.7 (0.8)	25.0 (0.7)	2.5
		288.3	60	25			
SAR	386.4	368.2 ^a^	60	28	27.8 (1.1)	21.9 (0.8)	21.3
		342.3	60	28			
SMM	281.2	156.0 ^a^	60	25	24.7 (0.7)	24.5 (0.5)	0.9
		215.1	60	25			
SM2	279.2	186.0 ^a^	60	28	66.9 (1.6)	64.0 (0.9)	4.3
		156.0	60	28			
SQ	301.3	156.0 ^a^	62	24	9.4 (0.3)	10.3 (2.9)	9.6
		91.7	62	44			
TIM	869.6	696.4 ^a^	120	63	78.1 (6.8)	76.1 (5.8)	2.5
		174.4	130	57			
TYL	916.6	174.3 ^a^	101	52	50.4 (2.5)	54.2 (2.4)	7.4
		772.6	101	41			
KIT	772.4	109.1 ^a^	90	78	87.3 (3.2)	82.0 (2.5)	6.1
		174.2	90	50			
TAM	494.5	192.2 ^a^	48	29	60.8 (3.6)	50.7 (1.4)	16.5
		119.2	48	55	52.6 (0.8)	55.7 (1.2)	5.9

^a^ Product ion for quantification. ^b^ Declustering potential. ^c^ Collision energy. ^d^ The relative abundance ratio of the identification ion intensity to the quantification ion intensity at 50 μg L^−1^. ^e^ Relative standard deviation. SRM, selected reaction monitoring.

**Table 2:** Linearity, recovery, and precision for the method in the chicken muscle sample.

Analyte	Linearity	Spiked Level (μg kg^−1^)			Intra-Day Recovery (%, *n* = 6)									Intra-Day RSD ^a^ (%, *n* = 6)									Inter-Day Recovery (%, *n* = 18)			Inter-Day RSD ^a^ (%, *n* = 18)		
	(*r*^2^)	I	II	III	I			II			III			I			II			III			I	II	III	I	II	III
ENR	0.9971	2	50	100	77.8	86.2	90.8	81.2	82.5	96.6	87.5	91.4	93.0	8.0	8.4	11	4.6	7.4	2.7	3.0	6.7	5.0	84.9	86.8	90.6	7.8	9.8	3.8
CIP	0.9994	2	50	100	69.0	77.2	82.5	69.8	67.6	77.4	76.6	70.8	78.2	8.7	6.7	10	9.1	7.3	14	6.4	6.5	2.2	76.2	71.6	75.2	13	10	5.2
SAR	0.9986	2	10	20	77.3	77.2	89.2	79.5	77.3	99.7	76.9	81.2	91.9	6.3	11	10	5.1	2.6	1.5	1.7	9.0	11	81.2	85.5	83.3	20	13	11
SMM	0.9986	2	50	100	67.5	78.1	79.0	75.4	75.6	79.4	74.7	79.6	81.8	5.3	3.8	5.3	2.4	5.5	4.6	5.1	2.1	2.2	74.9	76.8	78.7	8.9	2.9	4.6
SM2	0.9996	2	50	100	85.9	85.5	93.7	83.2	82.6	94.7	75.7	80.6	81.0	4.6	5.2	5.6	6.8	4.2	8.0	2.0	6.9	5.6	88.4	86.8	79.1	2.4	6.3	9.0
SQ	0.9994	2	50	100	68.8	67.9	77.4	69.7	69.2	68.4	70.2	69.1	70.7	4.3	1.1	3.9	3.7	2.5	6.3	2.0	1.9	0.8	71.4	69.1	70.0	7.7	6.3	6.4
TIM	0.9984	5	50	100	81.5	82.1	102.4	75.0	72.2	80.9	75.6	71.1	80.7	3.3	7.9	4.4	4.3	8.1	8.0	14	13	6.9	88.7	76.0	75.8	12	7.9	12
TYL	0.9992	5	50	100	80.2	67.3	80.4	80.3	67.2	75.8	79.3	68.8	67.9	7.6	6.5	4.3	3.3	4.3	5.5	5.6	5.2	7.2	76.0	74.4	72.0	12	15	12
KIT	0.9997	5	50	100	69.2	73.7	76.8	67.1	69.5	70.5	68.2	72.9	71.0	8.7	7.9	1.4	3.5	14	6.0	5.4	8.4	6.9	73.2	69.0	70.7	6.6	8.7	9.3
TAM	0.9998	2	50	100	74.4	69.1	80.4	73.9	72.7	73.3	80.4	75.2	74.7	5.7	16	1.3	8.3	12	7.9	5.8	8.4	4.8	74.6	73.3	76.8	10	8.4	6.5

^a^ Relative standard deviation. I, II, and III represent the three spiking levels of low, medium, and high concentrations, respectively.

**Table 3:** LOD and LOQ for the method and the MRL values of 10 drugs in chicken muscle.

Analyte	LOD ^a^	LOQ ^b^	MRLs ^c^ (μg kg^−1^)	
	(μg kg^−1^)	(μg kg^−1^)	China	European Union
ENR	0.5	2.0	100 ^d^	100 ^d^
CIP	0.5	2.0	100 ^d^	100 ^d^
SAR	0.5	2.0	10	NS ^f^
SMM	0.5	2.0	100 ^e^	100 ^e^
SM2	0.5	2.0	100	100 ^e^
SQ	0.5	2.0	100 ^e^	100 ^e^
TIM	2.0	5.0	75	75
TYL	2.0	5.0	200	100
KIT	2.0	5.0	100	NS ^f^
TAM	0.3	2.0	100	100

^a^ Limit of detection. ^b^ Limit of quantification. ^c^ Maximum residue limits. ^d^ Sum of enrofloxacin and ciprofloxacin. ^e^ All substances belonging to the sulfonamide group. ^f^ Not specified in chicken muscle.

**Table 4:** Comparison of the proposed method with the method reported in the literature.

Category	I	II
Compound (X)	FQs (3), SAs (3),	FAs (4), SAs (4), MCs (4), LINCs (2),
	MCs (3), and TAM	AGs (3),β-LACTs (3), TCs (3) and AMPR
Mobile phase	0.05% formic acid in ACN (A) and 0.05% formic acid in water (B)	50 mm ammonium formate in water at pH 2.5 (A) and ACN (B)
Extraction solvent	1% acetic acid in ACN	2% TCA aqueous solution: ACN (1:1, *v/v*)
Dilution times	10	10
LOD (μg kg^−1^)	0.3 (TAM)–2.0 (MCs)	0.1 (SAs)–20 (DSTR)
LOQ (μg kg^−1^)	2.0 (TAM–5.0 (MCs)	0.3 (SAs)–60 (DSTR)
Recovery (%)	67.1 (KIT)–96.6 (ENR)	53 (ENR)–99 (OXO)
Matrix effects (%)	−28.4 (SQ)–21.3 (ENR)	−99 (AMPR)–53 (DSTR)

I, The proposed method, II, The method reported in the literature [32], (x) = number of veterinary drugs, FQs, fluoroquinolones, SAs, sulfonamides, MCs, macrolides, LINCs, lincosamides, AGs, aminoglycisides, β-LACTs, β-lactams, TCs, tetracyclines, AMPR, amprolium, ACN, Acetonitrile, TCA, Trichloroacetic acid, LOD, Limit of detection, LOQ, Limit of quantification, TAM, tiamulin, DSTR, dihydrostreptomycin, KIT, kitasamycin, ENR, enrofloxacin, OXO, oxolinic acid, SQ, sulfaquinoxaline.

**Table 5:** Time of administration and time point slaughter for chickens.

**Days**	I	II	III	IV	V	VI	VII	VIII
**Drugs**	A	A + B	A + B + C	B + C	C	-	No. 2	No. 3

A: TIM 15 mg kg^−1^, SM2 50 mg kg^−1^, SMM 20 mg kg^−1^, SQ 100 mg·kg^−1^, B: ENR 8 mg kg^−1^, SAR 8 mg kg^−1^, C: TYL 700 mg kg^−1^, KIT 360 mg·kg^−1^, and TAM 40 mg·kg^−1^. No. 2: the second chicken, No. 3: the third chicken.
